# Atmospheric Electricity Influencing Biogeochemical Processes in Soils and Sediments

**DOI:** 10.3389/fphys.2019.00378

**Published:** 2019-04-16

**Authors:** Ellard R. Hunting, R. Giles Harrison, Andreas Bruder, Peter M. van Bodegom, Harm G. van der Geest, Andries A. Kampfraath, Michel Vorenhout, Wim Admiraal, Casper Cusell, Mark O. Gessner

**Affiliations:** ^1^School of Biological Sciences, University of Bristol, Bristol, United Kingdom; ^2^Biology Department, Woods Hole Oceanographic Institution, Woods Hole, MA, United States; ^3^Institute of Environmental Sciences, Leiden University, Leiden, Netherlands; ^4^Department of Meteorology, University of Reading, Reading, United Kingdom; ^5^Laboratory of Applied Microbiology, University of Applied Sciences and Arts of Southern Switzerland, Bellinzona, Switzerland; ^6^Freshwater and Marine Ecology, Institute for Biodiversity and Ecosystem Dynamics, University of Amsterdam, Amsterdam, Netherlands; ^7^MVH Consult, Leiden, Netherlands; ^8^Department of Experimental Limnology, Leibniz-Institute of Freshwater Ecology and Inland Fisheries, Stechlin, Germany; ^9^Department of Ecology, Berlin Institute of Technology, Berlin, Germany

**Keywords:** atmospheric electricity, bacterial respiration, biogeochemistry, Carnegie-curve, ions, redox potential

## Abstract

The Earth’s subsurface represents a complex electrochemical environment that contains many electro-active chemical compounds that are relevant for a wide array of biologically driven ecosystem processes. Concentrations of many of these electro-active compounds within Earth’s subsurface environments fluctuate during the day and over seasons. This has been observed for surface waters, sediments and continental soils. This variability can affect particularly small, relatively immobile organisms living in these environments. While various drivers have been identified, a comprehensive understanding of the causes and consequences of spatio-temporal variability in subsurface electrochemistry is still lacking. Here we propose that variations in atmospheric electricity (AE) can influence the electrochemical environments of soils, water bodies and their sediments, with implications that are likely relevant for a wide range of organisms and ecosystem processes. We tested this hypothesis in field and laboratory case studies. Based on measurements of subsurface redox conditions in soils and sediment, we found evidence for both local and global variation in AE with corresponding patterns in subsurface redox conditions. In the laboratory, bacterial respiratory responses, electron transport activity and H_2_S production were observed to be causally linked to changes in atmospheric cation concentrations. We argue that such patterns are part of an overlooked phenomenon. This recognition widens our conceptual understanding of chemical and biological processes in the Earth’s subsurface and their interactions with the atmosphere and the physical environment.

## Introduction

Concentrations of various chemical compounds in surface waters, soils and sediments have been observed to vary widely in both space and time, often detetable as diel (but also seasonal) fluctuations (e.g., Stockdale et al., 2009; Nimick et al., 2011; Smith et al., 2011). This variability is highly relevant for organisms that live within these spatio-temporally heterogeneous environments. While large mobile organisms interact at broader spatial scales, small and relatively immobile organisms like bacteria, fungi or nematodes can be expected to be particularly sensitive to fluctuations in their immediate electrochemical environment. Small organisms respond to changes in their physico-chemical environment with changes in metabolic activity and behavior (e.g., Fenchel, 2002; Vanreusel et al., 2010). Disposal of respiratory electrons is essential for organisms to sustain metabolic activity that drives ecosystem processes, including respiration and the recycling of organic matter and nutrients (Cho and Azam, 1988). The availability of molecules accepting respiratory electrons (i.e., redox conditions) can hence pose an important constraint on the metabolic activity of organisms in soils and sediments (Hayes and Waldbauer, 2006).

While many studies have improved our understanding of processes governing the Earth’s subsurface electrochemical environment, many observed variations remain difficult to reconcile with known drivers of electrochemical heterogeneity. Here, we briefly synthesize our understanding of drivers of Earth subsurface electrochemical variability, and present a novel conceptual foundation relating variations in atmospheric electricity (AE) to variations in the Earth’s electrochemical environment and to consequences for the microorganisms living therein. We present evidence supporting the proposed linkages and identify challenges for future research.

## Drivers of Spatio-Temporal Variability in Earth’s Subsurface Electrochemistry

Small-scale variation in the electrochemical properties of sediments and soils are mainly controlled by biotic influences. For instance, locomotive activity of invertebrates that rework soils and sediments (bioturbation) is a well-known driver of micro to millimeter-scale redox conditions in both soils and sediments (Tokida et al., 2007; Hunting et al., 2012). Bacterial metabolic activity, in particular, is considered to be mainly controlled by this small-scale variation (Newman and Banfield, 2002). Redox fluctuations are likely an important selective pressure on microbes with repercussions for community composition and activity (Pett-Ridge and Firestone, 2005), for instance by selecting for metabolically more flexible bacterial taxa (DeAngelis et al., 2010). In turn, bacteria can secrete redox-active exudates (e.g., flavins) to maintain favorable redox conditions (Hunting and Kampfraath, 2013; Markelova et al., 2018), or can use long-distance (>1 cm) electron transfer to connect spatially separate bio-electrochemical processes (Nielsen et al., 2010; Pfeffer et al., 2012). Photosynthesis also promotes fluctuations in redox-conditions by introducing oxygen into the upper layers of soils and sediment (Battin et al., 2003; Laursen and Seitzinger, 2004), resulting in a net diurnal increase of oxygen concentrations and a net nocturnal decrease caused by respiration.

While small-scale variations are mainly driven by biological processes (Masscheleyn et al., 1991; Hayes and Waldbauer, 2006), diel and seasonal fluctuations of concentrations for many chemical species relevant to microbial processes (e.g., denitrification and methanogenesis) are also often linked over large distances (Lee, 1977; Laursen and Seitzinger, 2004; Allen et al., 2007; Spencer et al., 2007; Rusjan and Mikoš, 2010; Bass et al., 2013). The occurrence of large-scale temporal fluctuations in a wide variety of ecosystems suggests large-scale abiotic processes are also relevant to soil, sediment and water electrochemical properties (Scholefield et al., 2005). Indeed, various abiotic drivers of spatial linkages and synchronized temporal variability in subsurface chemical concentrations and microbial activity have been identified. They include solar activity, groundwater flow, atmospheric pressure, lunisolar and tidal cycles, and gradients of the chemical potential of charge carriers (reviewed in Lanzerotti and Gregori, 1986; Tokida et al., 2007). In inland waters and terrestrial soils, charge separation in clay or other minerals, contaminants and ground-water flow have also been shown to influence the electrochemical environment (e.g., Revil and Jardani, 2013).

Despite the breadth of understanding of processes governing the Earth’s subsurface electrochemical environment and the consequences for organisms, the known drivers fail to explain all observed electrochemical variations. This is especially true for variations in the deeper layers (up to meters) of Earth’s surface (Vorenhout et al., 2011). For instance, while photosynthesis can be responsible for diel variation of redox-conditions in biofilms and surficial (<1 cm) soil and sediment layers (Battin et al., 2003; Laursen and Seitzinger, 2004), it unlikely affects deeper environments and associated organisms, since oxygen diffusion is slow and consumption by heterotrophs is fast (Laursen and Seitzinger, 2004). Here, we propose a new perspective based on the idea that variation in AE is an additional factor underlying cyclic variation in the electrochemistry and associated microbial communities and activities in the Earth’s subsurface environment.

## Conceptual Foundation of Relationships Between Atmospheric Electricity, Earth’s Subsurface Electrochemistry and Microbial Communities

Electrical properties of the near-surface atmosphere (e.g., ion concentrations and the atmospheric potential gradient) vary on daily and seasonal times scales (Israelsson and Tammet, 2001; Harrison, 2004). An atmospheric electric field is present even in fair-weather regions as a consequence of global electric current flows driven by thunderstorm regions (e.g., Rycroft et al., 2000; Haldoupis et al., 2017). Locally, environmental conditions, radioactive decay of radon, charges of aerosols, and atmospheric pollution may further contribute to variation in atmospheric electric conditions (Matthews et al., 2019). The combination of global and local variations in AE leads to variations over various spatial and temporal scales, with diel fluctuations being particularly important (Israelsson and Tammet, 2001). The universal diel pattern in vertical current and potential gradient is dominated by a minimum at around 04 Universal Time (UT) and a maximum at around 19 UT. This universal pattern is clearest in clean maritime air where aerosol pollution and other local sources of variation (e.g., diel fluctuations in radon concentration) are minimized. In contrast, over land diel patterns are more influenced by local variations in AE (Israelsson and Tammet, 2001; Harrison, 2004).

**FIGURE 1:**
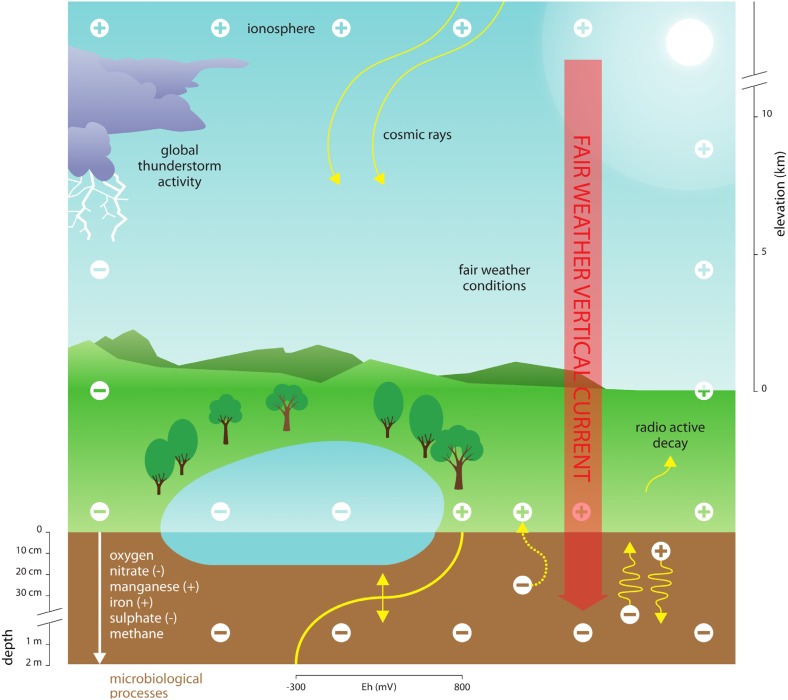
Conceptual diagram depicting the proposed link between atmospheric electricity (AE), Earth surface electrochemistry and microbial processes. Electrical variations of cations (+) in the atmosphere are governed by a variety of factors, including cosmic rays, variation in the ionosphere, radioactive decay of radon and other elements, global thunderstorm activity and solar radiation. Since the Earth surface is negatively charged (–), the resulting vertical current forces ions to move within soils and sediments. This includes the major ions required for microbial metabolic activities in anoxic environments. These changes in resource supply caused by electrical variation in the atmosphere can thus influence the spatial and temporal patterns of biogeochemical processes. The major terminal electron acceptors used in anoxic microbial metabolism can be either anions or cations (indicated by a – or + sign, respectively). Anions such as nitrate (NO_3_^-^) and sulfate (SO_4_^2-^) move toward the atmosphere, whereas cations such as iron (Fe^2+^) and manganese (Mn^2+^) move deeper into the soil or sediment. Free electrons produced by microbial metabolism at the Earth surface could also potentially be directed toward the atmosphere, as indicated by the curved dotted arrow.

Electric currents rely on movement of (small) ions in the atmospheric electric field, and typically range between 0.5 and 3.0 pA m^-2^ at the Earth surface interface, where the currents subsequently enter the Earth surface as part of the global electric circuit (Rycroft et al., 2008; Harrison, 2013). Other geophysical processes (e.g., groundwater flow) contribute to influencing the electrical properties of the Earth subsurface (Lanzerotti and Gregori, 1986; Wada and Umegaki, 2001; Revil et al., 2010). In soils, water bodies, and their sediments, currents induced by variations in AE likely influence the release of respiratory electrons and movement of ions, thereby critically affecting redox conditions with repercussions particularly for microorganisms. For instance, variations in AE could induce the vertical movement of charged terminal electron acceptors that are essential for microbial respiration (see Figure 1 for a conceptual diagram). Terminal electron acceptors relevant to microorganisms (e.g., NO_3_^-^, Mn_4_^3+^, and SO_4_^2-^) differ in size and charge, suggesting that they move at different speeds within Earth subsurface environments. Moreover, ion movement is influenced by the electrical conductivity of water, soils and sediments. Surface soil layers, for instance, typically have a conductivity of 0.1–2.0 dS/m (Rhoades and Corwin, 1981), which is low compared to seawater, for instance (∼ 4 S/m; e.g., Al-Shamma’a et al., 2004), and may impede long-distance (Revil et al., 2010) but not short-distance (μm – mm – cm) ion movement (Wada and Umegaki, 2001; Mann et al., 2005). Such variation in redox properties of soils and sediments driven by AE likely affects the ability of microbes to dispose of their respiratory electrons (Figure 1). To date, the consequences of variation in AE on the electrochemical properties of subsurface ecosystems and the organisms living therein remains entirely unexplored.

## Evidence for Linkages Between Atmospheric Electricity, Subsurface Electrochemistry and Microbial Metabolic Activity

### Laboratory Experiments

**FIGURE 2:**
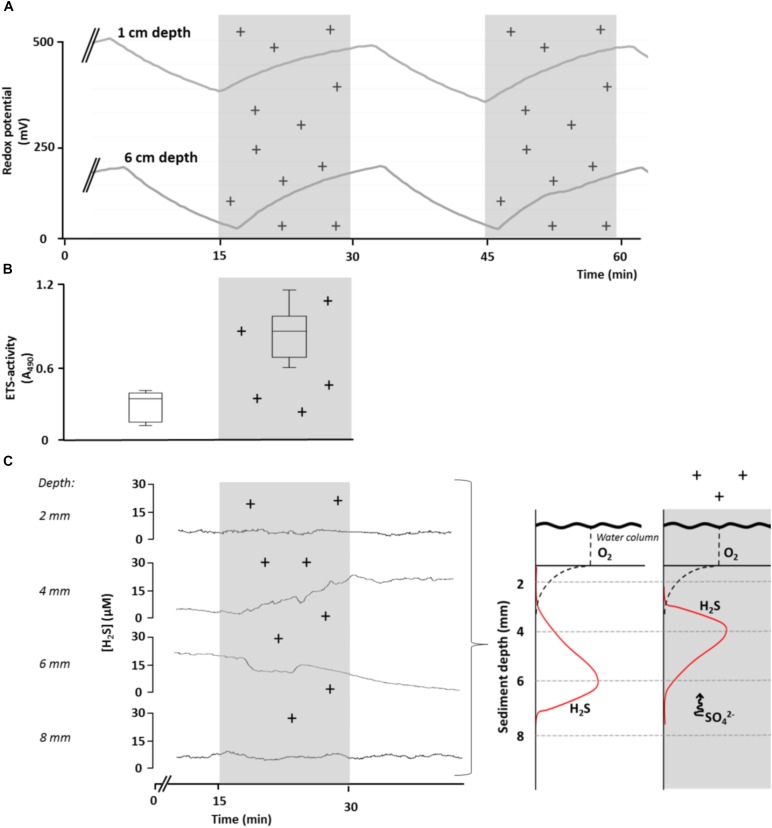
Effect of elevated levels of atmospheric cations on redox conditions, bacterial respiratory activity and H_2_S concentrations (sulfate reduction) in sediments of aquatic microcosms. **(A)** Redox potential, *E*_h_, was measured at 1 and 6 cm sediment depth in response to manipulated atmospheric cation concentrations. **(B)** Bacterial respiratory activity, measured as electron transport system (ETS) activity (expressed as relative absorption at 490 nm), was significantly lower in control microcosms than in microcosms in which the atmosphere was ionized for 24 h (*t*-test, *p* = 0.002, *n* = 6). **(C)** Changes in bacterial H_2_S concentration at different sediment depths in response to ionization of the overlying atmosphere (left panel), suggesting a shift in H_2_S production toward the surface as a result of upward SO_4_^2-^ movement (conceptually depicted in right panel). Shaded areas and (+) indicate periods of experimental ionization. A single time-series measurements is presented for clarity and considered representative of replicate (*n* = 12) runs.

We conducted several laboratory experiments to explore effects of variation in AE on sediment redox conditions and bacterial metabolism (for a description of the experimental approach see Supplementary Material S1). We found that sediment redox potential (*E*_h_) in aquatic microcosms evolved independently of sediment pH (max. change ± 0.1 units) or oxygen concentration (max. change ± 1% saturation), when exposing them to experimentally manipulated levels of atmospheric ion concentrations. In contrast, *E*_h_ gradually increased at different sediment depths, starting immediately when ionization began, then declined and quickly stabilized when disrupting ionization (Figure 2A). Control microcosms in which the overlying atmosphere was not ionized soon reached a redox equilibrium that remained constant throughout the experiment (data not shown). No effects on *E*_h_ in the sediment were found after exposing the microcosms to radiation [UV A, B, and C as well as photosynthetically active radiation (PAR) and infrared; data not shown]. Taken together, our empirical data shows that fluctuations of *E*_h_ in the microcosm sediment were independent of solar radiation but were strongly influenced by the manipulated shifts in ion concentrations in the overlying atmosphere. These findings provide clear evidence that variations in AE have potential to influence geochemical and microbial processes via alterations of *E*_h_.

We then tested the response of bacterial communities in sediments of microcosms to fluctuations in *E*_h_ induced by variations in AE laboratory conditions by increasing concentrations of ions in the overlaying atmosphere. We measured the respiration of the bacterial community as ETS activity in the upper sediment layer (<1 cm) after 1 day of exposure to atmospheric ionization and observed a two-fold increase (*t*-test, *p* < 0.05) compared to control microcosms (Figure 2B). However, since multiple bacterial processes can contribute to ETS activity, the cause of the increase remains uncertain.

To examine the issue further, we experimentally exposed aquatic microcosms to increased concentrations of ions in the overlaying atmosphere and assessed H_2_S concentrations in response to atmospheric ionization. We chose H_2_S concentration as the most informative response variable, since it results directly from SO_4_^-^ reduction. Moreover, since H_2_S does not carry a charge, any changes in H_2_S due to variation in AE can only result from changes in SO_4_^-^ reduction. We observed a gradual increase in H_2_S concentrations at 4 mm below the sediment surface, whereas H_2_S concentrations decreased at 6 mm below the sediment surface. The response in H_2_S concentrations after the start of ionization was slightly delayed (Figure 2C). These findings suggest that the depth of maximum SO_4_^2-^ concentrations shifted toward the sediment surface in response to ionization where the microbial community quickly responded by reducing SO_4_^2-^ to H_2_S.

### Field Observations

**FIGURE 3:**
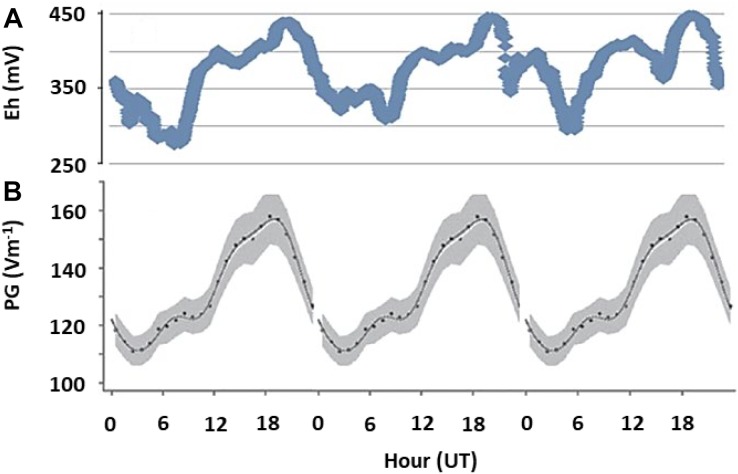
Fair weather diel fluctuations in sediment redox potential and AE. **(A)** Redox potential (E_h_) measured at 10 cm depth of a natural pristine sediment as a single run over 3 days in Lake Cadagno, an alpine lake in Switzerland, in October 2017. **(B)** The universal periodicity in atmospheric electrical properties (expressed as potential gradient, PG, between atmosphere and ground), which is visible around the globe during fair weather conditions (Harrison, 2013). All data is plotted against universal time, UT, and represent single time-series measurements. Peaks at 19 UT indicate that fluctuations in redox conditions are governed by global patterns in AE.

**FIGURE 4:**
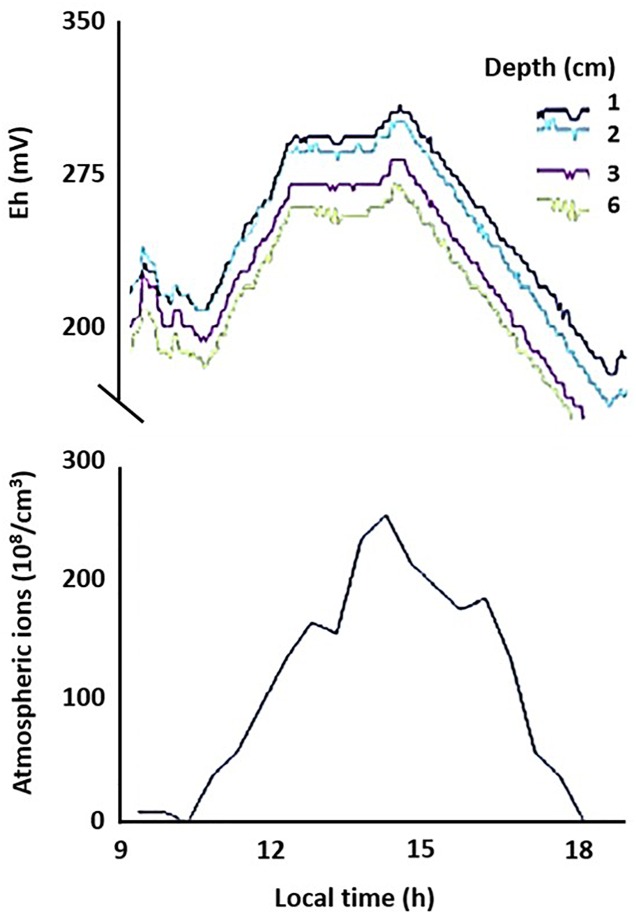
Coherence of temporal changes in net atmospheric cation concentrations and sediment redox conditions. Redox potential (Eh) was measured on 24 October 2013 at different depths (1, 2, 3, and 6 cm) in sandy sediment of a ditch in Netherlands. Data of this single run is plotted against local time (GMT +2).

To evaluate whether the links between AE and subsurface redox variations observed in microcosm experiments apply in realistic settings, we measured *E*_h_ in (1) an outdoor mesocosms facility containing no or different combinations of invertebrates causing bioturbation of surface sediments; (2) freshwater sediments at two distinct sites, and (3) soils at geographically distinct locations (see Supplementary Material S1 for details). In the shallow littoral zone of pristine Lake Cadagno in the Swiss Alps, we observed diel fluctuations in *E*_h_ following the universal cycle in atmospheric potential gradient, with peaks occurring at around 19 UT (Figure 3). In contrast, in a ditch experiencing urban pressure in Netherlands, the diel fluctuation was dominated by local influences, with a peak occurring at around 2–4 pm local time (Figure 4). However, the number of cations in the ground-level atmosphere also appeared to covary with sediment *E*_h_ (Figure 4).

**FIGURE 5:**
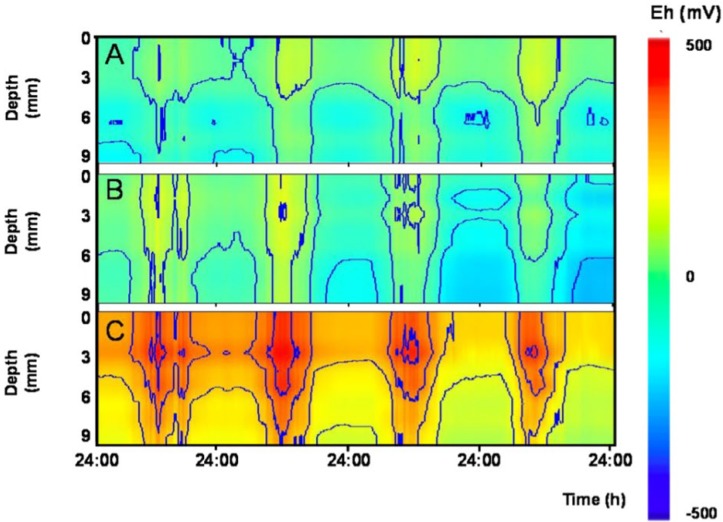
Fair weather diel rhythms in sediment redox potential outdoor mesocosms experiencing different levels of bioturbation. Contour plots show depth profiles (0–9 mm) redox potential (*E*_h_) over 4 days in three different mesocosms. Mesocosms contained different combinations of invertebrates known to rework surface sediment to various degrees: **(A)** no bioturbation: invertebrates lacking; **(B)** low level of bioturbation: *Tubifex* spp. and *Asellus aquaticus*; and **(C)** high level of bioturbation: *Gammarus pulex*, *Asellus aquaticus*, *Chironomus riparius*, *Tubifex* spp., and *Lumbriculus variegatus*.

**FIGURE 6:**
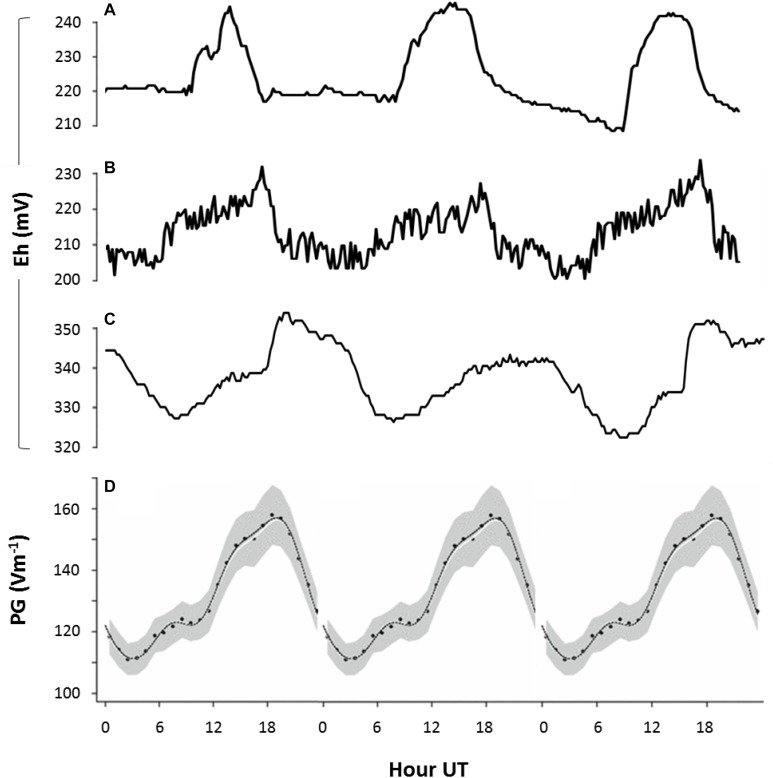
Fair weather diel rhythms in soil redox potential and AE. Redox potential (*E*_h_) measured in natural soils: **(A)** at 50 cm depth in Netherlands (52.2°N, 4.5°E; GMT + 2) between 1 and 3 May 2011, **(B)** at 50 cm depth in Netherlands (52.4°N, 6.1°E) between 1 and 3 July 2011, and **(C)** at 1 m depth in Bangladesh (23.8°N, 90.6°E; GMT + 6) between 27 and 30 March 2010. Lines represent measurements of single time series. **(D)** The universal periodicity in atmospheric electrical properties (expressed as potential gradient, PG, between atmosphere and ground) that is visible around the globe during fair weather conditions (Harrison, 2013). All data is plotted against, UT. Peaks at 19 UT (visible in panels **B**,**C**) indicate that fluctuations in redox conditions are governed by global variations in AE **(B,C)**, while peaks at 16 UT (visible in panel **A**) indicate that fluctuations in redox conditions are primarily governed by local variation in AE **(A)**.

Diel fluctuations in *E*_h_ in response to local variation in AE were also observed in sediments of freshwater outdoor mesocosms in Netherlands (Figure 5 and Supplementary Figure S1). Here, diel patterns were visible even in the presence of invertebrates reworking the upper sediment layers, and were more pronounced during fair-weather conditions than on cloudy days (Supplementary Figure S1). These diel rhythms were not observed to covary with other tested meteorological variables such as solar radiation, temperature and air pressure (data not shown). This finding indicates that the postulated link between AE and sediment redox potential persists also when major hydrological and geophysical processes (e.g., groundwater flow) are excluded. This supports our hypothesis that a direct link exists between subsurface *E*_h_ and AE. Interestingly, natural freezing of the top water layer in the mesocosms served as an unplanned experimental control, since the co variation between subsurface *E*_h_ and AE disappeared, probably as a consequence of poor conductive properties of ice (data not shown). Finally, *E*_h_ in soils at three distant sites also followed diel patterns in AE whose influence extended relatively deep into the soil (typically 50–100 cm; Figure 6).

These outdoor measurements suggest that variation in subsurface redox potential can follow both the universal diel cycle in the atmospheric potential gradient (Figure 6) and local sources of variation affecting atmospheric cation concentrations at ground level (<1 m). Together with our laboratory results, these field observations reveal that both global and local variations in AE influence redox conditions and microbial processes in soils and sediments, in which strong local influences on redox patterns can dominate in some locations.

## Implications and Perspectives

The findings from our laboratory experiments and field observations support the hypothesis that variation in AE can influence *E*_h_ in different soil and sediment matrices with consequences for microbial communities in these environments. The significance of this phenomenon in natural settings remains unclear, however, because persisting knowledge gaps impede a conclusive understanding of the causal relationships between AE and Earth’s electrical environment. Challenges for future research range from elucidating the relevant scales of the physical and chemical linkages to how these links directly or indirectly govern distinct groups of organisms.

How specific chemical species and organisms respond to changes in AE likely depends on the relative magnitude of a combination of various physical sources of variations. These include solar activity, groundwater flow, gradients of the chemical potential of charge carriers (reviewed in Lanzerotti and Gregori, 1986; Revil and Jardani, 2013), as well as electrochemical soil and sediment properties, including electrical resistance and the size and charge of terminal electron acceptors. Unraveling the absolute and relative roles of regional and global-scale drivers of variation in AE (Märcz and Harrison, 2003; Harrison, 2004) and redox potentials in water, sediments and soils would thus be a promising, though challenging research field. Earth subsurface electrochemistry varies in depth, and hence future work needs to assess to which extent variations in AE can translate to synchronized responses in Earth subsurface electrochemistry in relation to the conditions at different soil and sediment depths (e.g., moisture, conductivity, *E*_h_). Likewise, it is necessary to partition the role of AE in relation to other major drivers of Earth subsurface electrochemistry. This requires coordinated field experiments over a wide range of geographical locations to assess the significance on a global scale and the importance of local influences in superimposing universal rhythms.

The coupling between AE and subsurface electrochemistry observed in our field and laboratory studies also suggests that microorganisms in these environments are vulnerable to anthropogenic influences affecting variation in AE. In particular, anthropogenic pollution by smoke, sulfur dioxide and aerosols can affect AE (Retalis et al., 1991; Sheftel et al., 1994; Kamra and Deshpande, 1995). Our results suggest that such pollutants could have strong, though currently unknown, indirect effects on subsurface microorganisms and processes by affecting temporal patterns of AE. Furthermore, electrical pollution by high-voltage power lines (mostly operated with alternating currents) is a common local factor affecting variations in AE (Maruvada, 2012). The resulting static electric fields have been observed to trigger responses in a wide array of organisms, particularly behavioral responses in invertebrates (Petri et al., 2017; Schmiedchen et al., 2018). However, these studies were limited to flying insects and invertebrates on top of soils, and hence potential impacts of static electric fields on subsurface microorganisms and invertebrates went unnoticed. Nonetheless, power lines could influence soil and sediment communities and processes in at least two ways: First, shedding of ions provides a secondary source of pollution that may change direct current and ion transport in local environments with the consequences on microbial communities and processes described above. Second, strong variation in electric fields affect organisms using them for orientation (i.e., galvanotaxis or electrotaxis). Such behavioral responses have been observed for bacteria and invertebrates such as nematodes (Bespalov et al., 1996; Chrisman et al., 2016) and might further complicate the electrochemical environment in soils and sediments, which many organisms can alter (Traunspurger et al., 1997; Weerman et al., 2011; Hunting et al., 2013, 2015; Hunting and Kampfraath, 2013). Effects of local electrical pollution are readily amenable to tests in laboratory conditions by manipulating electric variables, but they can also be validated in natural settings (e.g., under power lines).

## Conclusion

Our results from experiments and field observations suggest that variation in AE can influence Earth’s subsurface chemistry and the microorganisms in subsurface environments. We have provided proof of evidence that variations in AE can cascade down to changes in sediment redox conditions with implications for microbial electron transport activities and biogeochemical processes such as SO_4_^-^ reduction. These insights widen our conceptual understanding of processes in water bodies, soils and sediments, and their overlooked links to AE. The coupling of AE and subsurface electrochemistry is likely relevant to a wide range of organisms, in particular those with electrotactic behavior such as many microbial and nematode species. The proposed concept that AE could serve as a sinus node that sets the pace of Earth’s biogeochemical heartbeat also presents many unknowns that call for pursuing diverse research avenues in the future.

## Author Contributions

EH conceived, designed, and coordinated the study. RH and AK were involved in the initial conception. EH, AB, MV, and CC collected the field data. HvdG and PvB participated in the design of the study. HvdG, EH, and AK participated in the design of the conceptual figure. EH performed the experiments and statistical analyses. EH, RH, and MG drafted the manuscript. WA, PvB, AB, and HvdG contributed significantly to the earlier drafts. All authors contributed to improve the earlier drafts of the manuscript.

## Conflict of Interest Statement

The authors declare that the research was conducted in the absence of any commercial or financial relationships that could be construed as a potential conflict of interest.

## References

[B1] Allen D. E. Dalal R. C. Rennenberg H. Meyer R. L. Reeves S. Schmidt S. (2007). Spatial and temporal variation of nitrous oxide and methane flux between subtropical mangrove sediments and the atmosphere. *Soil Biol. Biochem.* 39 622–631. 10.1016/j.soilbio.2006.09.013

[B2] Al-Shamma’a A. I. Shaw A. Saman S. (2004). Propagation of electromagnetic waves at MHz frequencies through seawater. *IEEE Trans. Antennas Propag.* 52 2843–2849. 10.1109/TAP.2004.834449

[B3] Bass A. M. O’Grady D. Berkin C. Leblanc M. Tweed S. Nelson P. N. (2013). High diurnal variation in dissolved inorganic C, δ13C values and surface efflux of CO2 in a seasonal tropical floodplain. *Environ. Chem. Lett.* 11 399–405. 10.1007/s10311-013-0421-7 23219386

[B4] Battin T. J. Kaplan L. A. Newbold J. D. Hansen C. M. (2003). Contributions of microbial biofilms to ecosystem processes in stream mesocosms. *Nature* 426 439–442. 10.1038/nature02152 14647381

[B5] Bespalov V. A. Zhulin I. B. Taylor B. L. (1996). Behavioral responses of *Escherichia coli* to changes in redox potential. *Proc. Natl. Acad. Sci. U.S.A.* 93 10084–10089. 10.1073/pnas.93.19.10084 8816755 PMC38340

[B6] Cho B. C. Azam F. (1988). Major role of bacteria in biogeochemical fluxes in the ocean’s interior. *Nature* 332 441–443. 10.1038/332441a0 19759822

[B7] Chrisman S. D. Waite C. B. Scoville A. G. Carnell L. (2016). *C. elegans* demonstrates distinct behaviors within a fixed and uniform electric field. *PLoS One* 11:e0151320. 10.1371/journal.pone.0151320 26998749 PMC4801214

[B8] Cusell C. Mettrop I. S. van Loon E. E. Lamers L. P. M. Vorenhout M. Kooijman A. M. (2015). Impacts of short-term droughts and inundations in species-rich fens during summer and winter: large-scale field manipulation experiments. *Ecol. Eng.* 77 127–138. 10.1016/j.ecoleng.2015.01.025

[B9] DeAngelis K. M. Silver W. L. Thompson A. W. Firestone M. K. (2010). Microbial communities acclimate to recurring changes in soil redox potential status. *Environ. Microbiol.* 12 3137–3149. 10.1111/j.1462-2920.2010.02286.x 20629704

[B10] Fenchel T. (2002). Microbial behavior in a heterogeneous world. *Science* 296 1068–1071. 10.1126/science.1070118 12004118

[B11] Haldoupis C. Rycroft M. Williams E. Price C. (2017). Is the “Earth-ionosphere capacitor” a valid component in the atmospheric global electric circuit? *J. Atmos. Sol. Terr. Phys.* 164 127–131. 10.1016/j.jastp.2017.08.012

[B12] Harrison R. G. (2004). The global atmospheric electrical circuit and climate. *Surv. Geophys.* 25 441–484. 10.1007/s10712-004-5439-8

[B13] Harrison R. G. (2013). The carnegie curve. *Surv. Geophys.* 34 209–232. 10.1007/s10712-012-9210-2

[B14] Hayes J. M. Waldbauer J. R. (2006). The carbon cycle and associated redox processes through time. *Philos. Trans. R. Soc. B Biol. Sci.* 361 931–950. 10.1098/rstb.2006.1840 16754608 PMC1578725

[B15] Hunting E. R. de Goeij J. M. Asselman M. van Soest R. W. M. van der Geest H. G. (2010). Degradation of mangrove-derived organic matter in mangrove associated sponges. *Bull. Mar. Sci.* 86 871–877. 10.5343/bms.2010.1001

[B16] Hunting E. R. Kampfraath A. A. (2013). Contribution of bacteria to redox potential (Eh) measurements in sediments. *Int. J. Environ. Sci. Technol.* 10 55–62. 10.1007/s13762-012-0080-4

[B17] Hunting E. R. Mulder C. Kraak M. H. S. Breure A. M. Admiraal W. (2013). Effects of copper on invertebrate–sediment interactions. *Environ. Pollut.* 180 131–135. 10.1016/j.envpol.2013.05.027 23747821

[B18] Hunting E. R. Vijver M. G. van der Geest H. G. Mulder C. Kraak M. H. Breure A. M. (2015). Resource niche overlap promotes stability of bacterial community metabolism in experimental microcosms. *Front. Microbiol.* 6:105. 10.3389/fmicb.2015.00105 25759686 PMC4338809

[B19] Hunting E. R. Whatley M. H. van der Geest H. G. Mulder C. Kraak M. H. Breure A. M. (2012). Invertebrate footprints on detritus processing, bacterial community structure, and spatiotemporal redox profiles. *Freshw. Sci.* 31 724–732. 10.1899/11-134.1

[B20] Israelsson S. Tammet H. (2001). Variation of fair weather atmospheric electricity at Marsta Observatory, Sweden, 1993–1998. *J. Atmos. Sol. Terr. Phys.* 63 1693–1703. 10.1016/S1364-6826(01)00049-9

[B21] Jeroschewski P. Steuckart C. Kuhl M. (1996). An amperometric microsensor for the determination of H2S in aquatic environments. *Anal. Chem.* 68 4351–4357. 10.1021/ac960091b

[B22] Kamra A. K. Deshpande C. G. (1995). Possible secular change and land-to-ocean extension of air pollution from measurements of atmospheric electrical conductivity over the Bay of Bengal. *J. Geophys. Res. Atmos.* 100 7105–7110. 10.1029/94JD03246

[B23] Lanzerotti L. J. Gregori G. P. (1986). *Telluric Currents: The Natural Environment and Interactions With Man-Made Systems. The Earth’s Electrical Environment*. Washington, DC: National Academy Press, 232–257.

[B24] Laursen A. E. Seitzinger S. P. (2004). Diurnal patterns of denitrification, oxygen consumption and nitrous oxide production in rivers measured at the whole-reach scale. *Freshw. Biol.* 49 1448–1458. 10.1111/j.1365-2427.2004.01280.x

[B25] Lee D. R. (1977). A device for measuring seepage flux in lakes and estuaries. *Limnol. Oceanogr.* 22 140–147. 10.4319/lo.1977.22.1.0140

[B26] Mann A. W. Birrell R. D. Fedikow M. A. F. De Souza H. A. F. (2005). Vertical ionic migration: mechanisms, soil anomalies, and sampling depth for mineral exploration. *Geochem. Explor. Environ. Anal.* 5 201–210. 10.1144/1467-7873/03-045

[B27] Märcz F. Harrison R. G. (2003). Long-term changes in atmospheric electrical parameters observed at Nagycenk (Hungary) and the UK observatories at Eskdalemuir and Kew. *Anna. Geophys.* 21 2193–2200. 10.5194/angeo-21-2193-2003

[B28] Markelova E. Parsons C. T. Couture R. M. Smeaton C. M. Madé B. Charlet L. (2018). Deconstructing the redox cascade: what role do microbial exudates (flavins) play? *Environ. Chem.* 14 515–524. 10.1071/EN17158

[B29] Maruvada P. S. (2012). Electric field and ion current environment of HVdc transmission lines: comparison of calculations and measurements. *IEEE Trans. Power Deliv.* 27 401–410. 10.1109/TPWRD.2011.2172003

[B30] Masscheleyn P. H. Delaune R. D. Patrick W. H. Jr. (1991). Effect of redox potential and pH on arsenic speciation and solubility in a contaminated soil. *Environ. Sci. Technol.* 25 1414–1419. 10.1021/es00020a008

[B31] Matthews J. C. Wright M. D. Clarke D. Morley E. L. Silva A. J. Bennett D. (2019). Urban and rural measurements of atmospheric potential gradient. *J. Electrostat.* 97 42–50. 10.1016/j.elstat.2018.11.006

[B32] Newman D. K. Banfield J. F. (2002). Geomicrobiology: how molecular-scale interactions underpin biogeochemical systems. *Science* 296 1071–1077. 10.1126/science.1010716 12004119

[B33] Nielsen L. P. Risgaard-Petersen N. Fossing H. Christensen P. B. Sayama M. (2010). Electric currents couple spatially separated biogeochemical processes in marine sediment. *Nature* 463 1071–1074. 10.1038/nature08790 20182510

[B34] Nimick D. A. Gammons C. H. Parker S. R. (2011). Diel biogeochemical processes and their effect on the aqueous chemistry of streams: a review. *Chem. Geol.* 283 3–17. 10.1016/j.chemgeo.2010.08.017

[B35] Petri A. K. Schmiedchen K. Stunder D. Dechent D. Kraus T. Bailey W. H. (2017). Biological effects of exposure to static electric fields in humans and vertebrates: a systematic review. *Environ. Health* 16:41. 10.1186/s12940-017-0248-y 28416002 PMC5393013

[B36] Pett-Ridge J. Firestone M. K. (2005). Redox fluctuation structures microbial communities in a wet tropical soil. *Appl. Environ. Microbiol.* 71 6998–7007. 10.1128/AEM.71.11.6998-7007.2005 16269735 PMC1287741

[B37] Pfeffer C. Larsen S. Song J. Dong M. Besenbacher F. Meyer R. L. (2012). Filamentous bacteria transport electrons over centimetre distances. *Nature* 491 218–221. 10.1038/nature11586 23103872

[B38] Retalis D. Pitta A. Psallidas P. (1991). The conductivity of the air and other electrical parameters in relation to meteorological elements and air pollution in Athens. *Meteorol. Atmos. Phys.* 46 197–204. 10.1007/BF01027345

[B39] Revil A. Jardani A. (2013). *The Self-Potential Method: Theory and Applications in Environmental Geosciences*. Cambridge: Cambridge University Press, 1–385. 10.1017/CBO9781139094252

[B40] Revil A. Mendonça C. A. Atekwana E. A. Kulessa B. Hubbard S. S. Bohlen K. J. (2010). Understanding biogeobatteries: where geophysics meets microbiology. *J. Geophys. Res. Biogeosci.* 115 10.1029/2009JG001065

[B41] Rhoades J. D. Corwin D. L. (1981). Determining soil electrical conductivity-depth relations using an inductive electromagnetic soil conductivity meter 1. *Soil Sci. Soc. Am. J.* 45 255–260. 10.2136/sssaj1981.03615995004500020006x

[B42] Rusjan S. Mikoš M. (2010). Seasonal variability of diurnal in-stream nitrate concentration oscillations under hydrologically stable conditions. *Biogeochemistry* 97 123–140. 10.1007/s10533-009-9361-5

[B43] Rycroft M. J. Harrison R. G. Nicoll K. A. Mareev E. A. (2008). An overview of earth’s global electric circuit and atmospheric conductivity. *Space Sci. Rev.* 137 83–105. 10.1007/s11214-008-9368-6

[B44] Rycroft M. J. Israelsson S. Price C. (2000). The global atmospheric electric circuit, solar activity and climate change. *J. Atmos. Sol. Terr. Phys.* 62 1563–1576. 10.1016/S1364-6826(00)00112-7

[B45] Schmiedchen K. Petri A. K. Driessen S. Bailey W. H. (2018). Systematic review of biological effects of exposure to static electric fields. Part II: invertebrates and plants. *Environ. Res.* 160 60–76. 10.1016/j.envres.2017.09.013 28963966

[B46] Scholefield D. Le Goff T. Braven J. Ebdon L. Long T. Butler M. (2005). Concerted diurnal patterns in riverine nutrient concentrations and physical conditions. *Sci. Total Environ.* 344 201–210. 10.1016/j.scitotenv.2005.02.014 15907518

[B47] Sheftel V. M. Chernyshev A. K. Chernysheva S. P. (1994). Air conductivity and atmospheric electric field as an indicator of anthropogenic atmospheric pollution. *J. Geophys. Res. Atmos.* 99 10793–10795. 10.1029/94JD00287

[B48] Smith L. Watzin M. C. Druschel G. (2011). Relating sediment phosphorus mobility to seasonal and diel redox fluctuations at the sediment–water interface in a eutrophic freshwater lake. *Limnol. Oceanogr.* 56 2251–2264. 10.4319/lo.2011.56.6.2251

[B49] Spencer R. G. Pellerin B. A. Bergamaschi B. A. Downing B. D. Kraus T. E. Smart D. R. (2007). Diurnal variability in riverine dissolved organic matter composition determined by in situ optical measurement in the San Joaquin River (California, USA). *Hydrol. Process.* 21 3181–3189. 10.1002/hyp.6887

[B50] Stockdale A. Davison W. Zhang H. (2009). Micro-scale biogeochemical heterogeneity in sediments: a review of available technology and observed evidence. *Earth Sci. Rev.* 92 81–97. 10.1016/j.earscirev.2008.11.003

[B51] Tokida T. Miyazaki T. Mizoguchi M. Nagata O. Takakai F. Kagemoto A. (2007). Falling atmospheric pressure as a trigger for methane ebullition from peatland. *Glob. Biogeochem. Cycles* 21 1–8.

[B52] Traunspurger W. Bergtold M. Goedkoop W. (1997). The effects of nematodes on bacterial activity and abundance in a freshwater sediment. *Oecologia* 112 118–122. 10.1007/s004420050291 28307367

[B53] Vanreusel A. Fonseca G. Danovaro R. Da Silva M. C. Esteves A. M. Ferrero T. (2010). The contribution of deep-sea macrohabitat heterogeneity to global nematode diversity. *Mar. Ecol.* 31 6–20. 10.1111/j.1439-0485.2009.00352.x

[B54] Vorenhout M. van der Geest H. G. Hunting E. R. (2011). An improved datalogger and novel probes for continuous redox measurements in wetlands. *Int. J. Environ. Anal. Chem.* 91 801–810. 10.1080/03067319.2010.535123

[B55] Wada S. I. Umegaki Y. (2001). Major ion and electrical potential distribution in soil under electrokinetic remediation. *Environ. Sci. Technol.* 35 2151–2155. 10.1021/es001335j 11414012

[B56] Weerman E. J. Van Der Geest G. H. Van Der Meulen M. D. Manders E. M. Van De Koppel J. Herman P. M. (2011). Ciliates as engineers of phototrophic biofilms. *Freshw. Biol.* 56 1358–1369. 10.1111/j.1365-2427.2011.02574.x

