# Supplementary material for: Atmospheric Electricity Influencing Biogeochemical Processes in Soils and Sediments
Source: Front Physiol. 2019 Apr 16;10:378. doi: 10.3389/fphys.2019.00378 (PMC6477044; doi:10.3389/fphys.2019.00378)
Supplement: Supplementary file 1 [file Table_1.pdf]

Supplementary material S1: Materials and Methods

Outdoor redox potential dynamics: Redox potentials (E_h) in soils at three locations at geographically distinct sites (Amsterdam, The Netherlands, 52.2°N, 4.5°E; Weerribben-Wieden, The Netherlands, 52.4°N, 6.1°E; and Araihazar, Bangladesh, 23.8°N, 90.6°E) were measured using permanently installed platinum E_h-electrodes connected to a Hypnos 3 data logger. Details on these devices and electrodes are described elsewhere[25,29]. E_h was recorded every 15 minutes, and corrected for the reference electrode and measured pH as described previously (Cusell et al., 2015). At one co-located site (Amsterdam, The Netherlands, 52.2°N, 4.5°E), the atmospheric ion concentration was measured using a cylindrical capacitor operating on the 'Gerdien-tube' principle (Alpha-lab Inc., Salt Lake City, UT, USA), calibrated to ambient conditions at midnight. Sediment redox potential dynamics were measured in a natural Alpine lake, Cadagno, Switzerland (46.5504° N, 8.7119° E), in October 2017, and in outdoor mesocosms that consisted of rectangular 90 L plastic tubes (L×W×H, 66 × 34 × 30 cm, respectively), containing ca. 40 L of rainwater and 18.5 L of sediment made of standardized garden soil (Baseline, Maxeda DIY, Diemen, The Netherlands) and quartz sand (0.1-0.5 mm; 62 Dorsilit, Eurogrit, Papendrecht, The Netherlands) mixed in a ratio of 5 L soil per 25 kg sand. These mesocosms were placed in concrete containers filled with water to buffer temperature fluctuations. Several invertebrate combinations were tested: A) a mesocosms without invertebrates; B) a mesocosm with the combination [*Tubifex* spp. and *Asellus aquaticus*]; and C) a mesocosm with the combination [*Tubifex* spp., *A. aquaticus*, *Chironomus riparius*, *Lumbriculus variegatus* and *Gammarus pulex*]. Invertebrates were added up to equal total quantities of 500 mg.DW/m2.

Bacterial respiratory activity in aquatic microcosms. Microcosms used to assess responses of bacterial respiratory activity to AE consisted of 400-mL glass jars (25.1 cm^2 surface area) containing 50.2 cm^3 sediment that was sourced from a pre-mixed bulk sediment composed of ignited quartz sand, 5% natural organic matter and a bacterial inoculum derived from sediments and water from a local wetland. Although we did not characterize the bacterial community in each microcosm, we assumed that this approach resulted in a sufficiently homogenous bulk sediment that would provide the replicate microcosms the same staring conditions and bacterial community. After gently adding 150 mL of water (Dutch Standard Water, DSW; a standardized synthetic analogue of common Dutch surface waters), the microcosms were left for 2 days to equilibrate. The atmosphere overlying the unsealed microcosms was subsequently manipulated using a standard 12 kV AC/DC high-voltage generator connected to a micro-tip needle to generate positively charged atmospheric ions (cations). Cation concentrations in the overlying atmosphere were increased to represent the net diel increase measured at a nearby wetland (i.e. 10^9 ions/cm^3), in which ion concentrations in the atmosphere were measured with a cylindrical capacitor as described above. Six microcosms were exposed for 24 h to an ionised atmosphere (n=6) and in six control microcosms at ambient conditions (n=6). Bacterial respiratory activity was assessed at the end of the 24 h incubation by determining electron transport system (ETS) activity in the surficial layers of the sediments in the microcosms. ETS activity was measured by following the reduction of 2-(p-iodophenyl)-3-(p-nitrophenyl)-5-phenyl tetrazolium chloride (INT) to formazan (INTF). In brief, 1 mL sediment was collected from the microcosms, vortexed with 1 mL of microcosm water and centrifuged for 5 s at 2000 *g*. Supernatants (400 μL) with suspended bacteria were subsequently assayed for ETS activity (Hunting et al., 2010).

H_2S concentration in microcosms. Microcosms used to determine H_2S production were made of polycarbonate and contained sulphate-rich sediment mixed with quartz sand. The overlying atmosphere was ionised as described above, except that ionisation was intermittent

with 15-min ON/OFF intervals. Microprofiles of O_2, H_2S and pH were measured with Unisense® x25 microsensors attached to a Unisense® multimeter (Jeroschewski et al., 1996). Given that the micromanipulator captured newly formed ions by its metal casting, microelectrodes were fixed in plastic guiders at fixed sediment depth to evaluate the evolution of H_2S production over time. Numerous runs were required to identify the depth where changes in H_2S primarily occurred, which indicated that H_2S production appeared most pronounced within the top 1 cm of the sediment. Repetitive runs were subsequently used to assess whether changes in H_2S production occurred in response to changes in the ion concentrations in the overlying atmosphere, in which we focused on the top 6 cm with 0.2 cm intervals. High-frequency noise in the electrode output corresponded with the frequency and amplitude of the high-voltage generator. Data are therefore presented as moving averages (period of 40), and as representative of repetitive runs. Redox potential (E_h) was measured during all microcosm incubations using Au-plated PCB E_h-electrodes permanently installed at 1 and 6 cm sediment depth and connected to a Hypnos 3 datalogger (Vorenhout et al., 2011). E_h was recorded every minute, and also corrected against a calomel reference electrode and measured pH.

References

Cusell C, Mettrop IS, van Loon EE, Lamers LPM, Vorenhout M, Kooijman AM 2015 Impacts of short-term droughts and inundations in species-rich fens during summer and winter: Large-scale field manipulation experiments. *Ecological Engineering*, 77, 127-138

Hunting ER, de Goeij JM, Asselman M, van Soest RWM, van der Geest HG. 2010 Degradation of mangrove-derived organic matter in mangrove associated sponges. *Bulletin of Marine Science,* 86(4), 871-877.

Jeroschewski P, Steuckart C, Kuhl M. 1996 An amperometric microsensor for the determination of H2S in aquatic environments. *Anal. Chem*. 68, 4351-4357.

Vorenhout M, van der Geest HG, Hunting ER. 2011 An improved datalogger and novel probes for continuous redox measurements in wetlands. *International Journal of Environmental and Analytical Chemistry,* 91(7-8), 801-810.
