# Supplementary material for: Atmospheric Electricity Influencing Biogeochemical Processes in Soils and Sediments
Source: Front Physiol. 2019 Apr 16;10:378. doi: 10.3389/fphys.2019.00378 (PMC6477044; doi:10.3389/fphys.2019.00378)
Supplement: Supplementary file 2 [file Data_Sheet_1.pdf]

Supplementary Figure S1: Natural fluctuation of sediment redox potentials during fair weather and during days with extensive cloud cover. a) Redox potential in the top (0-9mm: light grey to dark grey) layer of a ditch sediment during 4 consecutive days (3-11-2011 till 6-11-2011). **b)** Cloud cover (grey line) and solar radiation (black line) during the redox measurements were obtained from the Royal Netherlands Meteorological Institute (KNMI): https://data.knmi.nl. Distinct peaks within the diel rhythms of sediment redox potential correspond to periods of fair weather conditions. Outdoor mesocosms consisted of rectangular 90 L plastic tubs (L*W*H, 66 x 34 x 30 cm, 60 respectively), containing ca. 40 L of rainwater and 18.5 L of sediment made of standardized garden soil (Baseline, Maxeda DIY, Diemen, The Netherlands) and quartz sand (0.1-0.5 mm; 62 Dorsilit, Eurogrit, Papendrecht, The Netherlands) mixed in a ratio of 5 L soil per 25 kg sand. These mesocosms were placed in concrete containers filled with water to buffer temperature fluctuations. Sediment redox potential (Eh) profiles were used to characterize the sediment by recording Eh with permanently installed redox potential microelectrodes and a calomel reference electrode connected to a Hypnos data logger (Vorenhout et al., 2011)[1].

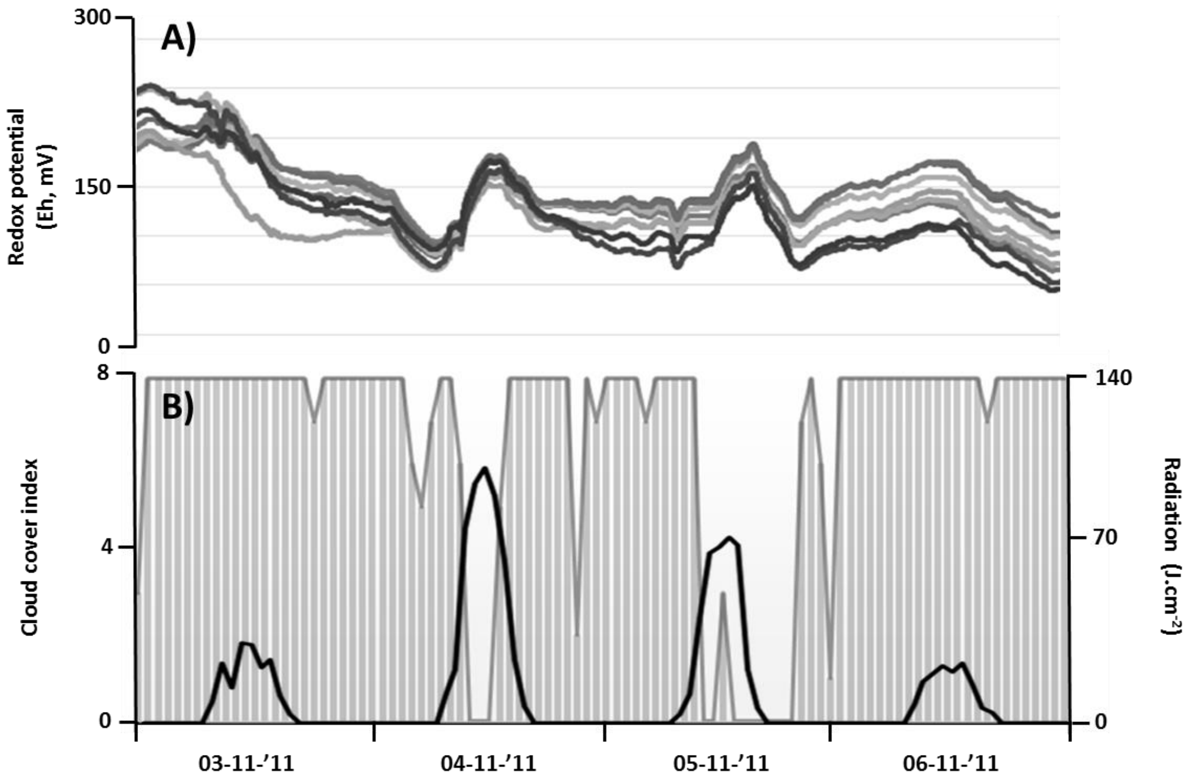

[1] Vorenhout, M., van der Geest, H. G., & Hunting, E. R. (2011). An improved datalogger and novel probes for continuous redox measurements in wetlands. *International Journal of Environmental and Analytical Chemistry*, *91*(7-8), 801-810.
